# Diseased muscles that lack dystrophin or laminin-α2 have altered compositions and proliferation of mononuclear cell populations

**DOI:** 10.1186/1471-2377-5-7

**Published:** 2005-04-07

**Authors:** Mahasweta Girgenrath, Christine A Kostek, Jeffrey Boone Miller

**Affiliations:** 1 Neuromuscular Biology & Disease Group, Boston Biomedical Research Institute, 64 Grove Street, Watertown MA 02472, USA

## Abstract

**Background:**

Multiple types of mononucleate cells reside among the multinucleate myofibers in skeletal muscles and these mononucleate cells function in muscle maintenance and repair. How neuromuscular disease might affect different types of muscle mononucleate cells had not been determined. In this study, therefore, we examined how two neuromuscular diseases, dystrophin-deficiency and laminin-α2-deficiency, altered the proliferation and composition of different subsets of muscle-derived mononucleate cells.

**Methods:**

We used fluorescence-activated cell sorting combined with bromodeoxyuridine labeling to examine proliferation rates and compositions of mononuclear cells in diseased and healthy mouse skeletal muscle. We prepared mononucleate cells from muscles of *mdx *(dystrophin-deficient) or *Lama2*^-/- ^(laminin-α2-deficient) mice and compared them to cells from healthy control muscles. We enumerated subsets of resident muscle cells based on Sca-1 and CD45 expression patterns and determined the proliferation of each cell subset *in vivo *by BrdU incorporation.

**Results:**

We found that the proliferation and composition of the mononucleate cells in dystrophin-deficient and laminin-α2-deficient diseased muscles are different than in healthy muscle. The *mdx *and *Lama2*^-/- ^muscles showed similar significant increases in CD45^+ ^cells compared to healthy muscle. Changes in proliferation, however, differed between the two diseases with proliferation increased in *mdx *and decreased in *Lama2*^-/- ^muscles compared to healthy muscles. In particular, the most abundant Sca-1^-^/CD45^- ^subset, which contains muscle precursor cells, had increased proliferation in *mdx *muscle but decreased proliferation in *Lama2*^-/- ^muscles.

**Conclusion:**

The similar increases in CD45^+ ^cells, but opposite changes in proliferation of muscle precursor cells, may underlie aspects of the distinct pathologies in the two diseases.

## Background

In diseased skeletal muscle, damaged myofibers can sometimes be replaced or repaired by mononucleate muscle precursor cells that can commit to myogenesis and fuse to form multinucleate myofibers. In some muscle diseases, progressive loss of muscle function may be due at least in part to a decreasing rate of repair that becomes insufficient to replace lost myofibers. The mononucleate cells in skeletal muscle include the relatively abundant muscle satellite cells and their myoblast progeny, as well as infiltrating inflammatory cells and the much less numerous muscle stem cells [1-4]. In this study, we examine how different subsets of muscle-derived mononucleate cells are affected in the diseased skeletal muscles of laminin-α2-deficient (*Lama2*^-/-^) and dystrophin-deficient (*mdx*) mice. We examined these two diseases because both the extracellular laminin-α2 and the intracellular dystrophin proteins interact with the dystroglycan complex of membrane proteins, yet the pathologies of the two diseases are significantly different.

Mice lacking laminin-α2 due to mutations in the *Lama2 *gene have greatly shortened lifespan, poor muscle growth, and poor muscle regeneration compared to normal littermates [5,6]. Laminin-α2 is a subunit of the basement membrane component known as merosin (or laminin 2/4) and is highly expressed in skeletal muscle [7,8]. *Lama2*^-/- ^mice are a model for the severely disabling human disease, primary laminin-α2-deficient Congenital Muscular Dystrophy Type 1A [9,10], which is also known as merosin-deficient congenital muscular dystrophy [11,12].

The *mdx *mice carry a point mutation in the dystrophin gene that leads to loss of functional dystrophin protein. Muscle pathology in 2–4 month old *mdx *mice is characterized by widespread muscle fiber degeneration and regeneration [13]. Unlike *Lama2*-null mice, *mdx *mice have almost normal life spans and their muscles show evidence of continuing, successful regeneration [14,15]. The human disease, Duchenne muscular dystrophy, is also caused by mutations in the dystrophin gene.

For this study, we classified mononucleate cells derived from skeletal muscles based on expression of two cell surface proteins: Sca-1 and CD45. Sca-1 (Stem cell antigen 1) was first described as a hematopoietic stem cell marker [16] and subsequently found to be expressed by a rare subset of the mononucleate cells in skeletal muscles [17-19]. CD45 is a cell surface tyrosine phosphatase found on all nucleated cells of hematopoietic origin and is also found on rare muscle-derived cells [1]. The expression patterns of these two proteins define four subsets of cells. The double-negative subset includes muscle satellite cells and their myoblast progeny [20]. The double-positive subset includes cells that appear to be multi-potential stem cells [1]. The Sca-1^-^/CD45^+ ^subset includes infiltrating inflammatory cells; and different subsets of this heterogeneous group of cells are found in dystrophin-deficient and laminin-α2-deficient muscles [21-27]. The Sca-1^+^/CD45^- ^subset includes cells with myogenic and endothelial potential [28].

Previous studies suggested that satellite cells and their myoblast progeny have higher than normal proliferation in *mdx *muscles [15,31], but proliferation rates in *Lama2*^-/- ^muscles had not been well studied. In addition, it was known that injury induced by cardiotoxin leads to an increase in the CD45^+ ^cells in skeletal muscles [3,4]. No previous studies, however, had examined how dystrophin-deficiency and laminin-α2-deficiency might alter the proliferation and composition of different subsets of muscle-derived mononucleate cells. In this study, therefore, we examined proliferation rates and compositions of the Sca-1 and CD45 defined subsets of skeletal muscle cells. Our results show that dystrophic muscles (both *mdx *and *Lama2*^-/-^) have a similarly increased percentage of CD45^+ ^cells compared to muscles of wild-type mice. Furthermore, we found that most cell subsets in *mdx *muscles *in vivo *had increased proliferation relative to normal muscles, whereas most cell subsets in *Lama2*^-/- ^muscles had lower than normal proliferation. The contrasting proliferation profiles of the different subsets of mononucleate cells in dystrophin-deficient and laminin-α2-deficient muscles may explain aspects of the distinct pathologies of the two diseases.

## Methods

### Mice, breeding, and genotyping

For dystrophin-deficient muscles, we used mice of the C57BL/10ScSn-*Dmd*^*mdx*^/J genotype (*mdx*); and as a normal muscle control for the dystrophin-deficient mice, we used C57BL/10ScSn mice (Jackson Laboratory, Bar Harbor ME). For laminin-α2-deficient muscles, we used mice that carry a targeted LacZ insertion that inactivates the *Lama2 *gene which encodes laminin-α2 [5,32]. This targeted allele is termed *dy-W *[5]. Heterozygous *Lama2*^*dy*-*W*/+ ^mice were a gift of Dr. Eva Engvall (Burnham Institute, La Jolla CA). Breeding of these heterozygotes in our laboratory was used to obtain homozygous, laminin-α2-null (*Lama2 *^-/-^) progeny, as well as heterozygous (*Lama2*^+/-^) and normal (*Lama2*^+/+^) littermates. Littermates were genotyped by analysis of the *Lama2*^*dy*-*W *^targeted mutation and the wild-type allele by PCR of DNA obtained from tail biopsies as described [5]. Muscles from normal littermates served as controls for *Lama2*^-/- ^muscles. The *Lama*^*2dy*-*W*/*dy*-*W *^homozygotes express only very small amounts of a truncated laminin-α2 that lacks domain VI [6], and they have a severe neuromuscular disease in which about most mice die by 6 weeks of age [32]. All animal experiments were reviewed and approved by the Institutional Animal Care and Use Committee at the Boston Biomedical Research Institute.

### Muscle cell isolation and fluorescence-activated cell sorting

To prepare cells for FACS, *Lama2*^-/- ^(n = 3 at 3 weeks old), *mdx *(n = 2 at 4 weeks old or n = 3 at 7.5 week old), and the appropriate normal control mice (n = 2 for 4 week old controls and n = 3 for 3 week and 7.5 week old controls) were injected intra-peritoneally with 100 μl of 5-bromo-2'-deoxyuridine (BrdU) (BrdU Flow Kit, BD Biosciences) solution (1 mg/ml in PBS) at 24 hours and again at 16 hours before sacrifice. Limb muscles were dissected, cleaned, and thoroughly minced. The tissue was digested in 0.2% Pronase (Calbiochem) in HBSS at 37°C for one hour and successively passed through nylon mesh filters with cut-offs of 100 μm, 40 μm, and 10 μm. After the final filtering, cells were re-suspended in 3% heat-inactivated fetal bovine serum in PBS. Biotinylated Sca-1 antibody (E13-161.7) and phycoerythrin (PE)-conjugated CD45 antibody (clone 30-F11) (both from Pharmingen, San Diego CA) were added to the cells at a concentration of 2 μg/ml, and the cells were incubated on ice for one hour. Cells were washed in PBS and incubated with the secondary detecting reagent, allophycocyanin (APC)-streptavidin, at 1 μg/ml for 20 minutes on ice. After three additional washes in PBS, the cells were fixed for 30 minutes with a DPBS buffer containing 4% paraformaldehyde and the detergent saponin, then washed and stored overnight in staining buffer (from the BrdU flow kit) supplemented with 3% fetal bovine serum.

The following day, cells were refixed as above, treated with 300 μg /ml DNase for one hour at 37°C, incubated for 20 minutes with fluorescein isothiocyanate (FITC) conjugated anti-BrdU antibody at a 1:50 dilution of the stock solution provided with the kit, and stained with 20 μl of a DNA binding dye, 7-amino-actinomycinD (7-AAD) (BrdU flow kit) for 1 hour. Separate aliquots of cells were stained with appropriate isotype control antibodies and used as negative controls. Flow cytometric analysis was performed using a dual-laser cell sorter (FACSCalibur, BD Biosciences) to determine the distribution and proliferation dynamics of the muscle-derived cells. Cell Quest Pro software (BD Biosciences) was used for data acquisition and analysis. Debris was first gated out by analyzing particle morphology based on side and forward scatter. *In vivo *proliferation was determined by analyzing the percent of all the cells that incorporated BrdU. Also, we determined the percent of cells within each sub-population that were BrdU positive.

### Statistical analyses

All data are expressed as mean ± standard deviation. Comparison among groups was done by analysis of variance (ANOVA) and individual differences between pairs was determined by unpaired two-tailed t-test performed with the Instat program (v2.03, GraphPad, San Diego CA). Statistical analyses were done either using raw data (prior to conversion to percentages) or using arcsin-transformed percentage data [33]. Differences among the disease groups or between diseased and normal controls was considered significant when p ≤ 0.05.

## Results

We used FACS analysis to identify subsets of the mononucleate cells that reside in skeletal muscle and then determined how the distribution of cells across the subsets was affected by disease. We identified the cell subsets based on expression or lack of expression of two cell surface markers: CD45 and Sca-1. Thus, we partitioned the muscle-derived cells into four subsets: Sca-1^+^/CD45^+^, Sca-1^+^/CD45^-^, Sca-1^-^/Cd45^+ ^and Sca-1^-^/CD45^-^. Cells for sorting were obtained from muscles of *mdx *mice and their 10ScSn normal controls at 4 weeks and 7.5 weeks after birth and from muscles of *Lama2*^-/- ^and normal littermates at 3 weeks after birth. The respective myopathies are well in progress at these times [31,32]. It was not possible to analyze older *Lama2*^-/- ^mice for Sca-1 and CD45 expression, as most died by 5–6 weeks of age and the small muscles did not consistently provide sufficient cells for the necessary FACS analyses.

**Figure 1:**
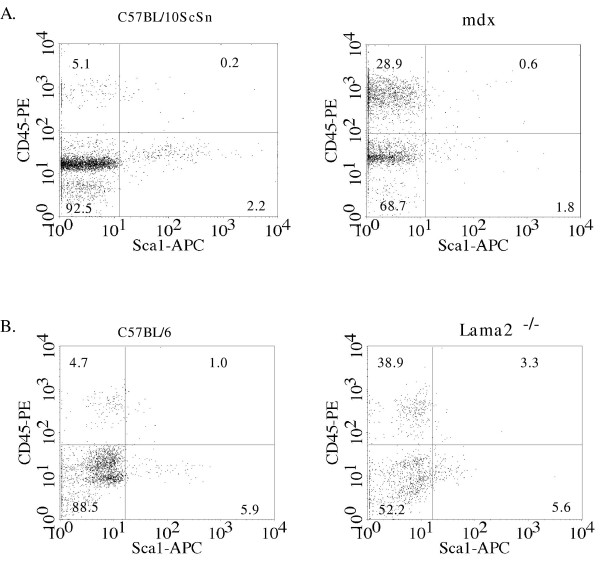
The composition of mononucleate cells was altered in *Lama2*^-/- ^and *mdx *diseased muscles compared to healthy muscles. Mononucleate cells were prepared from muscles of *Lama2*^-/- ^(3 week old), *mdx *(7.5 weeks old), and age- and strain-matched control mice and analyzed by FACS for expression of Sca-1 and CD45. **A**. Scatter-plots of a representative FACS study of the different cell subsets defined by Sca-1 and CD45 expression patterns in normal C57BL/10ScSn (left) and *mdx *(right) muscles. **B**. Scatter-plots of a representative FACS study of the different cell subsets in normal C57BL/6J (right) and *Lama2*^-/- ^(left) muscles. Significant differences were observed in the distribution of cells between the wild type and disease conditions (see Fig. 2 and Table 1 for quantitative and statistical analyses). As also noted in the text, *mdx *muscles contained several-fold more mononucleate cells per gram of tissue than *Lama2*^-/- ^muscles.

**Figure 2:**
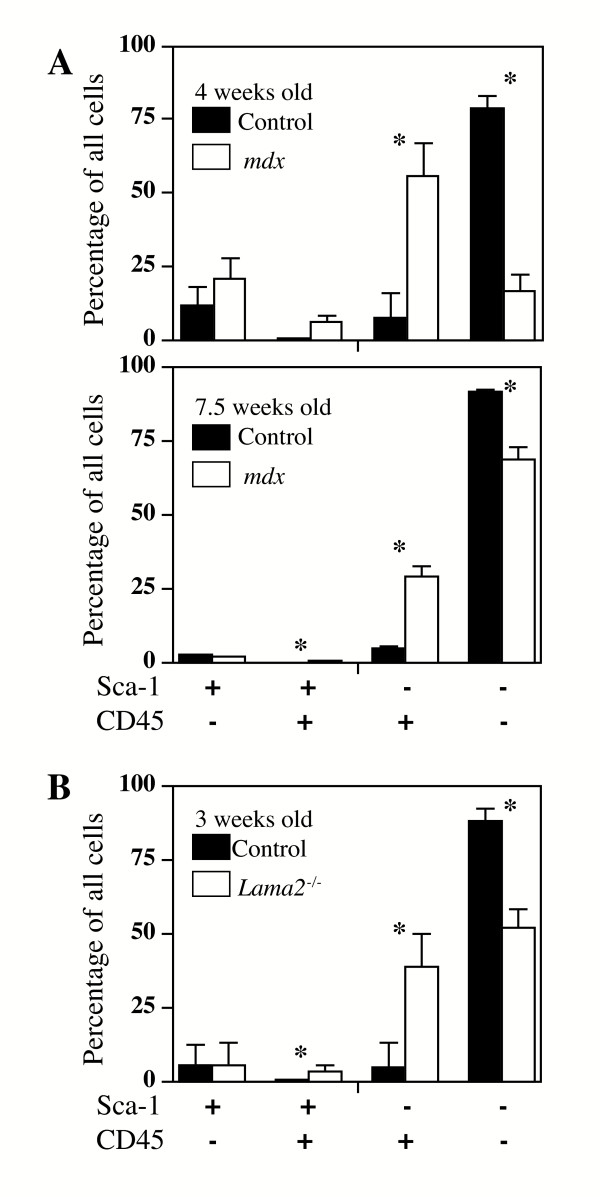
The composition of mononucleate cells was altered in diseased *mdx *and *Lama2*^-/- ^compared to control muscles. As in Fig. 1, mononucleate cells were prepared from muscles of *Lama2*^-/- ^(3 week old), *mdx *(4 weeks or 7.5 weeks old), and appropriate age- and strain-matched control mice and analyzed by FACS for expression of Sca-1 and CD45. **A**. Quantitative analysis of the cell subsets defined by Sca-1 and CD45 expression in *mdx *and normal control muscles showed that the percentage of cells in the two CD45^+ ^subsets was signficantly higher in *mdx *muscles at both 4 weeks and 7.5 weeks of age. **B**. Similarly, in *Lama2*^-/- ^muscles, the percentage of cells in the two CD45^+ ^subsets was also significantly higher than in normal healthy muscle. See text for statistical analyses. As also noted in the text, *mdx *muscles contained several-fold more mononucleate cells per gram of tissue than *Lama2*^-/- ^muscles. Error bars represent standard deviation with n = 3. * indicates that diseased and healthy control samples are significantly different at p < 0.05 (see Table 1 for quantitative and statistical analyses).

We found that cells from muscles of the two types of normal control mice that we used (C57Bl/10ScSn at 4 and 7.5 weeks old for *mdx *and C57BL/6 at 3 weeks old for Lama 2^-/-^) had similar distributions of cells across the four cell subsets (Figs. 1 and 2; Table 1). In all healthy muscles, the majority (>75% of the total) of mononucleate cells were negative for both Sca-1 and CD45. As noted [20], satellite cells and their myoblast progeny form multinucleate myotubes in culture, are Sca-1^-^/CD45^-^, and thus are included in this double-negative subset. We confirmed this result, because we found that (i) the double-negative cells at the time of isolation also expressed CD34, as expected for quiescent satellite cells [2]; (ii) >98% of the double-negative cells expressed desmin, an intermediate filament protein found in proliferating satellite cells, after 3 days in culture; and (iii) the double-negative cells were able to form myotubes in culture when switched to low serum medium (not shown). The rarest mononucleate cells (<1.5% of the total) in healthy muscles were those that expressed both Sca-1 and CD45. The remaining two cell subsets were more abundant than the double-positive cells, but still much less abundant than the double-negative cells. Cells that expressed Sca-1 but not CD45 amounted to 2.5 – 12% of the total in healthy muscles; and cells that expressed CD45 but not Sca-1 amounted to 4.7 – 8.3% of the total. The two Sca-1^+ ^subsets were more abundant in the 3 and 4 week old than in the 7.5 week old healthy muscles.

**Table 1:** Patterns of expression of Sca-1 and CD45 by mononucleate cells derived from healthy and diseased skeletal muscles.

		Percentage of cells with indicated expression pattern^1^			
Age	Genotype	Sca-1^+^/CD45^-^	Sca-1^+^/CD45^+^	Sca-1^-^/CD45^+^	Sca-1^-^/CD45^-^

3 weeks	C57BL/6	5.9 ± 3.5	1.0 ± 0.4	4.7 ± 1.7	88.0 ± 5.3
	*Lama2*^-/-^	5.6 ± 1.8	3.3 ± 0.4	38.9 ± 15.9	52.2 ± 15.6
		P > 0.1	P < 0.03*	P < 0.01*	P < 0.01*
4 weeks	C57BL/10	12.0 ± 6.7	1.2 ± 0.2	8.3 ± 2.2	78.6 ± 4.3
	*mdx*	21.0 ± 7.4	6.4 ± 2.4	55.8 ± 11.3	16.7 ± 6.2
		P > 0.1	P = 0.09	P < 0.03*	P < 0.01*
7.5 weeks	C57BL/10	2.5 ± 0.6	0.2 ± 0.1	5.1 ± 0.2	92.0 ± 0.6
	*mdx*	1.8 ± 0.5	0.8 ± 0.3	28.9 ± 3.6	68.5 ± 4.1
		P > 0.1	P = 0.03*	P < 0.01*	P < 0.01*

^1^Data are presented as ave. ± SD with n = 3 for all except 4 week old values where n = 2.

Unpaired, two-tailed t-tests (see Methods) were used to compare values from each pair of healthy and diseased muscles that were of the same age and expression pattern. P values are shown below the pair of tested values. P ≤ 0.05 was considered significant and indicated by an asterisk.

We next found that the distribution profiles of cells from dystrophin-deficient and laminin-α2-deficient muscles differed from those of cells from normal muscles, with the diseased muscles showing a marked increase in the CD45^+ ^cell subsets (Figs. 1 and 2, Table 1). The changes in the percentage of cells in each subset were similar for cells derived from *Lama2*^-/- ^and *mdx *muscles.

In particular, the rarest subset of double-positive CD45^+^/Sca-1^+ ^cells composed a significantly higher percentage of the mononucleate cells in diseased muscles (Figs. 1 and 2; Table 1). The percentage of double-positive cells was increased 3–5X in diseased compared to healthy muscles. As one example, the double-positive cells amounted to 3.3 ± 0.4% of the total cells from diseased Lama2^-/- ^muscles, but only 1 ± 0.4% of the cells from healthy C57BL/6 muscles (n = 3, P < 0.03).

The subset of CD45^+ ^cells that did not express Sca-1, which was moderately abundant in healthy muscle and includes inflammatory cells, also accounted for a higher percentage of the total mononucleate cells in diseased muscles than in normal muscles (Figs. 1 and 2; Table 1). In particular, the Sca-1^-^/CD45^+ ^cells accounted for a 5 – 8X higher percentage of the mononucleate cells in diseased than in healthy muscles. As one example, the 7.5 week old *mdx *muscles had a 5.7-fold increase in Sca-1^-^/CD45^+ ^cells compared to healthy control muscle (28.9 ± 3.6% of the cells in *mdx *samples *vs*. 5.1 ± 0.2% in control muscles; n = 3, P < 0.01). These increased percentages reflect the increased numbers of inflammatory cells that are known to occur in the skeletal muscles of these disease models [21-27]. These Sca-1^-^/CD45^+ ^inflammatory cells are known to be heterogeneous (*e. g*., macrophages, eosinophils, neutrophils, B-cells, T-cells; all of which are Sca-1^-^/CD45^+^); and the subsets of inflammatory cells that are present differ between dystrophin-deficient and laminin-α2-deficient muscles [21-27,29,30]. Because these different subsets of inflammatory cells have been well-characterized in dystrophin-deficient and laminin-α2-deficient muscles [21-27], we did not re-examine this heterogeneity for this study. The increased percentage of CD45^+ ^cells in diseased muscles was accompanied by a significantly decreased percentage of cells in the most abundant double-negative subset, whereas the percentage of cells in the Sca-1^+^/CD45^- ^subset was unchanged by disease (Figs. 1 and 2, Table 1).

Thus, in both dystrophin-deficient and laminin-α2-deficient diseased muscles, the double negative cells, which included satellite cells and their myoblast progeny, became relatively less abundant, whereas cells expressing the hematopoietic marker CD45 became relatively more abundant. As in previous studies, however, the absolute number of Sca-1^-^/CD45^- ^cells (muscle precursor cells) obtained from a given amount of *mdx *muscle was considerably larger than the number obtained from the same amount of control muscle, whereas fewer muscle precursor cells were obtained from *Lama2*^-/- ^than control muscles. For example, in one experiment using muscles from 5–6 week old *mdx *and control mice, healthy control muscles yielded 1.3 ± 0.4 × 10^5 ^double-negative cells per gram (the number of cells that can be obtained decreases with increasing age), whereas *mdx *muscles yielded many more at 4.8 ± 3.0 × 10^5 ^double-negative cells per gram (ave. ± SD, n = 4). In contrast, in an experiment using muscles from 3 week old *Lama2*^-/- ^and control mice, we obtained 2.6 ± 0.6 × 10^5 ^double-negative cells per gram of control muscle, but only 1.6 ± 0.3 × 10^5 ^double-negative cells per gram of *Lama2*^-/- ^muscle (ave. ± SD, n = 3). Thus, the similar increases in the percentage of CD45^+ ^cells in the two diseases occurred despite the different directions of change in total cell numbers.

**Figure 3:**
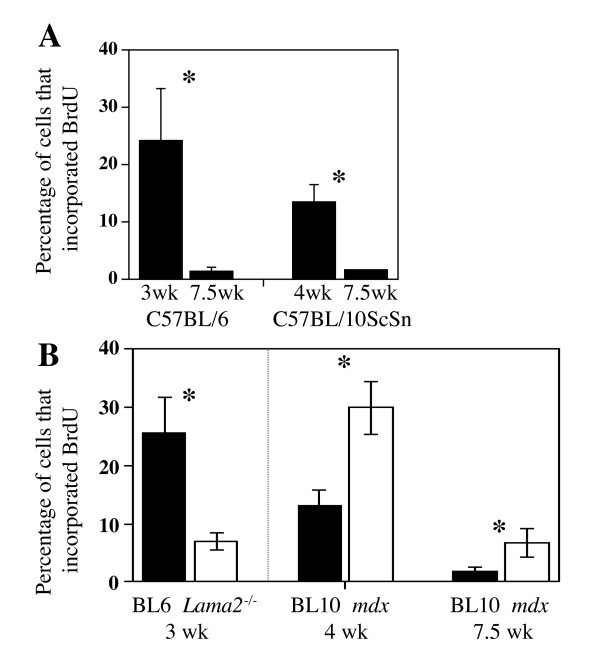
The percentage of cells that incorporated BrdU decreased with increased age and was lower in *Lama2*^-/- ^muscles, but higher in *mdx *muscles, than in normal control muscles. **A**. The percentage of the total population of mononucleate muscle cells that incorporated BrdU was decreased as postnatal development proceeded. In muscles of two control strains (C57Bl/6J, the control for *Lama2*^-/- ^muscles; and C57BL/10ScSn, the control for *mdx *muscles), there was a similar several-fold decrease in BrdU incorporation from 3–4 weeks to 7.5 weeks after birth, which is consistent with the slowing of muscle growth found during this period. **B**. The percentage of all cells that incorporated BrdU was significantly lower in 3 week old *Lama2*^-/- ^muscles than in normal control muscles of the same age. In contrast, the percentage of all that incorporated BrdU was signifcantly higher in 4 week old and 7.5 week old *mdx *muscles than in normal control muscles of the same age. Error bars represent standard deviation with n = 3. * indicates diseased and healthy control samples differ significantly at P < 0.05.

**Figure 4:**
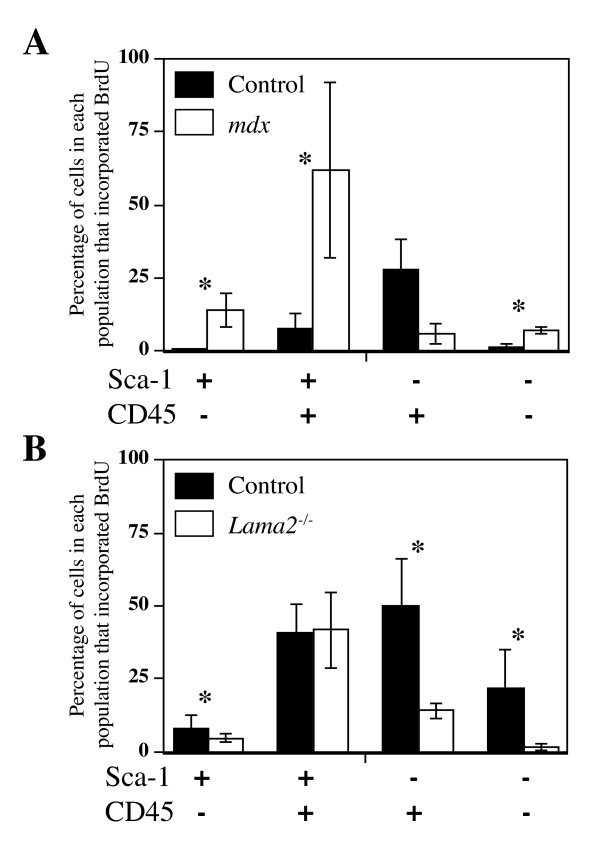
In most cell subsets, the percentage of cells that incorporated BrdU was lower in *Lama2*^-/- ^muscles, but higher in *mdx *muscles, than in healthy control muscles. **A**. In all cell subsets except Sca-1^-^/CD45^+^, the percentage of cells that incorporated BrdU during 24 h *in vivo *was significantly increased in *mdx *cells compared to normal cells (see text for statistics). **B. **The percentage of cells that incorporated BrdU in *Lama2*^-/- ^muscles was significantly decreased compared to normal muscles in all subsets of *Lama2*^-/- ^cells with the exception of the rare double-positive Sca-1^+^/CD45^+ ^subset in which incoporation was similar in diseased and healthy muscles (see text for statistics). Error bars represent standard deviation with n = 3. * indicates that the value for the *mdx *or *Lama2*^-/- ^muscles differed significantly from the corresponding control at P < 0.05.

Though both *mdx *and *Lama2*^-/- ^muscles showed similar increases in CD45^+ ^cells, the two diseases showed opposite changes in mononucleate cell proliferation. In muscles of the two control strains (C57Bl/6J, the control for *Lama2*^-/- ^muscles; and C57BL/10ScSn, the control for *mdx *muscles), there was a similar several-fold decrease in BrdU incorporation from 3–4 weeks to 7.5 weeks after birth, which is consistent with the slowing of muscle growth found during this postweaning period (Fig. 3A). At both 4 weeks and 7.5 weeks after birth, BrdU incorporation was significantly higher in *mdx *than in control muscles of the same age (Fig. 3B). In *mdx *muscles at 7.5 weeks after birth, for example, the percentage of all mononucleate cells that incorporated BrdU during 24 h *in vivo *was 6.9 ± 2.6%, which was significantly higher than 1.9 ± 1.1% that incorporated BrdU in the 10ScSn control muscles of the same age (n = 3, p < 0.05) (Fig. 3B). Consistent with this general increase of proliferation in *mdx *muscles, three of the four Sca-1/CD45 subsets of muscle-derived *mdx *cells showed increased BrdU incorporation, indicating a greater number of proliferating cells compared to normal controls. The exception was the Sca-1^-^/CD45^+ ^cell subset which had decreased BrdU incorporation in *mdx *compared to normal muscles (Fig. 4A).

In contrast, at 3 weeks after birth, a much lower percentage of cells incorporated BrdU in *Lama2*^-/- ^muscle than in normal muscle (Fig. 3B) (6.8 ± 1.7% for *Lama2*^-/-^*vs*. 23.5 ± 10.0% for normal. n = 3, p < 0.05). Consistent with this general decrease in proliferation, three of four Sca-1/CD45 subsets of muscle-derived *Lama2*^-/- ^cells showed decreased BrdU incorporation, indicating fewer proliferating cells compared to normal controls (Fig. 4B). The exception in this case was the double-positive Sca-1^+^/CD45^+ ^cell subset which had approximately the same BrdU incorporation in *Lama2*^-/- ^muscles as in normal muscles (Fig. 4B).

## Discussion

We found that the composition and proliferation dynamics of mononucleate cells were significantly different in the diseased muscles of *mdx *and *Lama2*^-/- ^mice than in normal healthy muscles. Dystrophin-deficient *mdx *muscles and laminin-α2-deficient *Lama2*^-/- ^muscles showed similar changes in cell compositions, with CD45^+ ^cells accounting for a significantly higher percentage of the total in diseased than in healthy muscles. In contrast, cells in *mdx *and *Lama2*^-/- ^muscles showed opposite changes in proliferation kinetics, with most cell subsets showing increased BrdU incorporation in *mdx *muscles, but decreased incorporation in *Lama2*^-/- ^muscles.

With the recognition that adult skeletal muscles contain rare cells with stem cell properties, a number of markers, including Sca-1, CD34, CD45, Bcl-2, and c-kit, have been used alone or in combination to identify and purify the cells [1,18,19,28,34-37]. In our study, we subdivided muscle-derived stem cells on the basis of expression or lack of expression of Sca-1, which is commonly used to purify hematopoietic stem cells, and CD45, which is a pan-hematopoietic cell marker. Neither Sca-1 nor CD45 is expressed by muscle satellite cells and their myoblast progeny (muscle precursor cells) [20], whereas muscle-derived cells with hematopoietic potential are almost all CD45^+ ^[1,20].

Though the distribution of cells among the four cell subsets did not significantly change during early postnatal (3 to 7.5 weeks) development in normal muscles (compare controls in Figs. 2A and 2B), there was a significant shift in the distribution profile in response to muscle disease. Specifically, diseased muscles had a large increase in the percentage of cells in both CD45^+ ^subsets, *i. e*., Sca-1^+^/CD45^+ ^and Sca-1-/ CD45^+ ^(Fig. 2, Table 1). The double-positive Sca-1^+^/CD45^+ ^cells were likely putative stem cells with hematopoietic potential [1], whereas the Sca-1^-^/CD45^+ ^cells included the heterogeneous group of inflammatory cells. The increase in CD45^+ ^cells was accompanied by a decrease in the double-negative Sca-1^-^/CD45^- ^cells, which included satellite cells and their myoblast progeny. Though the double-negative cells were usually the most abundant subset, we found that the Sca-1^-^/CD45^+ ^inflammatory cells became the most abundant subset in younger four week old *mdx *muscles, which is consistent with the rapid myofiber degeneration at this age.

A previous study showed that double-positive Sca-1^+^/CD45^+ ^cells also increase during regeneration of injured muscle and that a small proportion of the double-positive cells from injured muscle, unlike those from uninjured muscles, give rise to myogenic cells in culture [3,4]. Diseased muscles tend to undergo extensive degeneration followed by attempted regeneration. At least initially, regeneration is largely successful in *mdx *muscles but abortive in *Lama2*^-/- ^muscles [5,15,31]. Because the double-positive Sca-1^+^/CD45^+ ^cells increased in both types of diseased muscle (this study), it is likely that, as in injured muscles [3,4], these cells play a role in attempted muscle regeneration.

In contrast to the similar increases in CD45^+ ^cells in both types of diseased muscles, the mononucleate cells in *mdx *and *Lama-2*^-/- ^muscles showed opposite changes in proliferation *in vivo*, with proliferation increased in *mdx *muscles but decreased in *Lama-2*^-/- ^muscles compared to healthy control muscles.

Total cell proliferation in *mdx *muscles was known to be elevated relative to healthy controls [31]. Here we examined proliferation in subsets of the muscle mononucleate cells and found that, consistent with the increase in overall proliferation, a higher than normal percentage of cells in three of the four *mdx *subsets incorporated BrdU. Both the double-negative cells, which included satellite cells, and the double-positive cells with hematopoietic potential, showed increased proliferation. Only cells of the Sca-1^-^/CD45^+ ^subset failed to show increased BrdU incorporation in *mdx *muscles; and these were likely to be mostly inflammatory cells (*e. g*., macrophages, eosinophils, neutrophils, T-cells; all of which are Sca-1^-^/CD45^+^) which do not have hematopoietic stem cell potential but are known to migrate into and thus accumulate in diseased muscles [1,21-27]. The increased percentage of proliferating *mdx *cells *in vivo *is likely due to the altered environment in *mdx *muscles which includes increased levels of growth factors; increased extracellular matrix proteins such as collagen, fibronectin, and laminin which can promote myoblast proliferation; and perhaps decreased levels of growth inhibitors [38-42]. Whether cells in different subsets respond to the same or different growth regulators remains to be determined.

In *Lama2*^-/- ^muscles, overall BrdU incorporation was significantly decreased compared to that in healthy control muscles. BrdU incorporation was decreased in every *Lama2*^-/- ^cell subset except the rare double-positive Sca-1^+^/CD45^+ ^subset that includes cells with hematopoietic potential [1]. The decrease could have arisen if, relative to normal cells, the *Lama2*^-/- ^cells had longer cell cycles or lower probability of initiating replication from G_0_, if there was more cell death among dividing than non-dividing *Lama2*^-/- ^cells [43], or if some combination of these occurred. The relative lack of *Lama2*^-/- ^muscle cell proliferation could in part explain why regeneration is relatively unsuccessful in laminin-α2-deficient muscles [5], though other mechanisms such as increased apoptosis of both myoblasts and newly formed myofibers are also likely to be important.

Laminin-α2 is expressed in skeletal muscle myoblasts and myotubes [44] where it promotes the survival of satellite cells *in vivo *and *in vitro *as well as myoblast fusion and myotube formation [5,45,46]. We found that the most abundant, double-negative subset of muscle cells, which includes muscle precursor cells, showed significantly less BrdU incorporation in *Lama2*^-/- ^muscles. It is likely, therefore, that laminin-α2 not only promotes survival of myotubes as shown previously [45,46] but also influences division of muscle precursor cells as early as three weeks after birth, which is a time when extensive myofiber growth occurs in normal muscles. A recent study has demonstrated that laminin-2 (α2, β1, γ1) improves proliferation of an epithelial cell line through an integrin/ERK pathway [47]. A similar mechanism could occur in skeletal muscle cells, because merosin, of which laminin-α2 is a component, influences the level of expression and localization of α7β 1D integrin at the sarcolemma [46] and a lack of laminin-α2 could, therefore, disrupt merosin/integrin-mediated signals that regulate proliferation.

Pathogenesis follows distinct courses in *Lama2*^-/- ^and *mdx *mice, as well as in humans with corresponding diseases [48]. Nonetheless, CD45^+ ^cells are similarly increased in both mouse diseases. The role of the double-positive Sca-1^+^/CD45^+ ^cells in muscles needs to be defined, perhaps by tracing fates of their progeny; and the likely heterogeneity within the cell subsets we studied should be explored. For example, one study with a specific mAb suggested that satellite cells are <15% of the mononucleate cells in healthy, post-weaning skeletal muscles [49], suggesting that there might be heterogeneity within the double-negative Sca-1^-^/CD45^- ^cell subset which we and others [3] found to contain more than half of the mononucleate cells and to contain satellite cells. Some differences between studies might be expected because assessment of cell type percentages likely depends on the particular enzymes used for tissue dissociation, the antibodies and staining protocols used, and the selection of fluorescent cut-off limits for cells sorting. Despite these caveats, the general agreement between our study of healthy *vs*. diseased muscles and an earlier study [3] of healthy *vs*. cardiotoxin-injured muscles provides confidence in the conclusion that the CD45^+ ^cell subsets become relatively more abundant in both injured muscles and in the two types of diseased muscles analyzed here.

Further experiments in which particular cell subsets have their fates determined by lineage tracking or are experimentally increased or decreased, perhaps by specific growth factors or conditional ablation, could determine how the changes in cell subsets that we observed here may affect pathogenesis. The Sca-1^+^/CD45^- ^cell subset, for example, has been reported to contain cells with myogenic and endothelial lineage potentials [28]. Inhibition of apoptosis ameliorates myopathology and produces a several-fold increase in lifespan of *Lama2-null *mice [50], and could affect mononuclear cell subsets. That not only Sca-1^-^/CD45^- ^cells (which included satellite cells), but also other cell subsets, showed opposite changes of proliferation in *Lama2*^-/- ^and *mdx *muscle, suggests that growth requirements may be shared among the different subsets of cells. In addition, the relatively poor proliferation of *Lama2*^-/- ^cells may be one of the mechanisms underlying the lack of successful regeneration in *Lama2*^-/- ^muscles. The comparative significance to *Lama2*^-/- ^pathogenesis of poor proliferation *vs*. other mechanisms such as increased apoptosis remains to be defined.

## Conclusion

The similar increases in CD45^+ ^cells, but opposite changes in proliferation of muscle precursor cells, may underlie aspects of the distinct pathologies in dystrophin-deficiency and laminin-α2-deficiency.

## Competing interests

The author(s) declare that they have no competing interests.

## Authors' contributions

MG and CAK carried out all of the experimental studies. MG and JBM conceived the study, carried out statistical analyses and drafted the manuscript.

## Pre-publication history

The pre-publication history for this paper can be accessed here:


